# Identification and characterization of novel abdominal and pelvic brown adipose depots in mice

**DOI:** 10.1080/21623945.2022.2133415

**Published:** 2022-10-19

**Authors:** Ana M. Mesa, Theresa I. Medrano, Vijay K. Sirohi, William H. Walker, Richard D. Johnson, Sergei G. Tevosian, Angie M. Adkin, Paul S. Cooke

**Affiliations:** a Department of Physiological Sciences, University of Florida, Gainesville, FL, USA; b Department of Obstetrics, Gynecology and Reproductive Sciences, University of Pittsburgh and Magee-Womens Research Institute, Pittsburgh, PA, USA

**Keywords:** UCP1, thermogenesis, ureter, uterus, muscle

## Abstract

Brown adipose tissue (BAT) generates heat through non-shivering thermogenesis, and increasing BAT amounts or activity could facilitate obesity treatment and provide metabolic benefits. In mice, BAT has been reported in perirenal, thoracic and cranial sites. Here, we describe new pelvic and lower abdominal BAT depots located around the urethra, internal reproductive and urinary tract organs and major lower pelvic blood vessels, as well as between adjacent muscles where the upper hind leg meets the abdominal cavity. Immunohistochemical, western blot and PCR analyses revealed that these tissues expressed BAT markers such as uncoupling protein 1 (UCP1) and CIDEA, but not white adipose markers, and β3-adrenergic stimulation increased UCP1 amounts, a classic characteristic of BAT tissue. The newly identified BAT stores contained extensive sympathetic innervation with high mitochondrial density and multilocular lipid droplets similar to interscapular BAT. BAT repositories were present and functional neonatally, and showed developmental changes between the neonatal and adult periods. In summary, several new depots showing classical BAT characteristics are reported and characterized in the lower abdominal/pelvic region of mice. These BAT stores are likely significant metabolic regulators in the mouse and some data suggests that similar BAT depots may also exist in humans.

## Introduction

Brown adipose tissue (BAT) has attracted extensive recent scientific interest due to its characteristic ability to produce heat by non-shivering thermogenesis, a process that consumes calories and could potentially be harnessed to treat human obesity and associated metabolic conditions [1,2]. BAT contains extensive mitochondria expressing uncoupling protein 1 (UCP1) that generates heat and increases overall metabolic activity, with a concomitant dissipation of energy [1].

Temperature regulation through non-shivering thermogenesis in BAT is critical in neonatal mammals. Both rodents and humans have a large interscapular BAT (iBAT) depot during early life [3], and iBAT was first described in human foetuses in 1902 [4]. iBAT persists throughout life in rodents, but in humans regresses with age and is lost in adults. This finding led to the long-term consensus during the early and middle 20^th^ century that BAT was not important in humans. Although Heaton [5] subsequently reported that BAT was detectable histologically in adult and even elderly humans, BAT attracted only limited interest for many years.

Approximately a decade ago, several imaging studies stimulated interest in this field by demonstrating extensive and highly active BAT depots in thoracic and neck regions of adult humans [6–8]. Critically, the size and activity of BAT depots were increased by cold exposure [8,9] and varied seasonally [10]. In addition, correlations between leanness and BAT volumes were documented in humans, further emphasizing the importance of BAT in metabolic regulation [11].

Recent findings using a human clinical database of over 50,000 patients demonstrated that the presence and abundance of BAT correlate with lower incidences of cardiometabolic diseases, type 2 diabetes and other conditions. The presence of BAT was also associated with improved blood glucose, triglyceride and high-density lipoprotein values [12], indicating that BAT has metabolic benefits over and above weight regulation.

In addition to the long-known iBAT and perirenal BAT depots in mice, recent studies identified new upper thoracic/head/neck BAT depots [13–15]. BAT is present in the subscapular region, near the dorsal neck of the scapula, in the cervical region of the head, and surrounding the thoracic (descending) aorta [15]. Mo et al [13] described supraclavicular BAT depots in mice with histological, molecular and functional attributes of a classic BAT depot, and a recent report from this group described a new BAT depot in the deep neck region [16]. Zhang et al [14] recently used a labelled glucose analogue and PET/CT imaging to visualize BAT depots in mice using the same methodology employed in human subjects. In addition to confirming the presence of BAT depots in thoracic, cervical and axillary regions, they also noted an intense label uptake in the midline region above and below the kidney that is a signature of BAT, which they designated as the ventral spinal BAT depot.

Mice are the most widely used model for studies of both brown and beige adipose tissue. Beige adipose tissue is a third type of adipose tissue that is distinct from BAT and white adipose tissue (WAT) that contains varying amounts of adipocytes with functional and structural characteristics of BAT interspersed among the WAT [1]. Genetic manipulations in mice have provided major insights into BAT physiology [17], and overexpression or deletion of proteins, such as PRDM16 in mice, has increased understanding of BAT development and function [18]. Therefore, the extent and distribution of mouse BAT are important because this is the primary animal model for this field.

During the development of two new transgenic mouse models with altered androgen receptor function [19], we examined the internal reproductive organs of these animals and serendipitously discovered a previously undescribed depot of BAT in the vicinity of these organs. We then reasoned that there might be additional BAT depots in the pelvic/lower abdominal area, and we were able to identify three other new BAT depots in this region that were not associated with reproductive organs. Here, we document the presence and functionality of these previously unknown BAT depots in mice. These depots express classical histological, molecular and functional features of BAT, and necessitate an extensive re-evaluation of our understanding of BAT distribution in the mouse.

## Results

### Identification of new BAT depots in the pelvic and lower abdominal regions of adult mice

**Figure 1.**
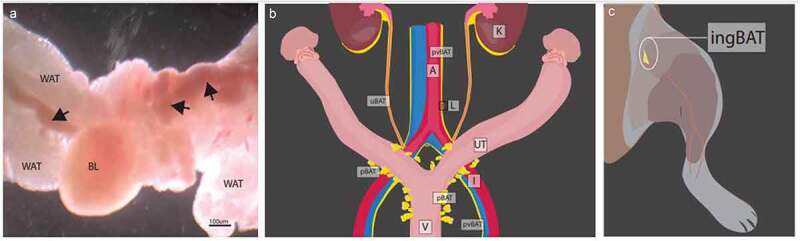
Visualization and localization of new BAT depots. (a) Pelvic brown adipose tissue (pBAT) in gonadal white adipose tissue (WAT) of an adult male mouse visualized with a dissecting microscope using transmitted light and darkfield microscopy. BAT is indicated by the arrows and can be differentiated from surrounding gonadal WAT by its darker colouration and less translucent appearance. (b) Location and appearance of the pBAT, perivascular BAT (pvBAT) and ureter BAT (uBAT) depots in female mice. (c) Location of the inguinal BAT (ingBAT) at the junction between the lower leg and the abdominal wall. BAT is shown in yellow. BL = bladder; WAT = white adipose tissue; K = kidney; UT = uterus; A = abdominal aorta; I = external iliac; L = lumbar lymph node; V = vagina.

When reproductive and urinary tract organs were removed and examined with a dissecting microscope using transmitted light and darkfield microscopy for an unrelated study [19], tissue that was subsequently determined to be BAT in these regions, which we term pelvic BAT (pBAT), could be clearly differentiated from surrounding WAT (Figure 1a) due to its dark reddish-brown colour and lack of an iridescent sheen that is characteristic of WAT. It was not possible to differentiate BAT in situ from the extensive surrounding WAT that comprises the gonadal fat pad when these tissues were examined using incident light.

In females, pBAT is found lateral to the vagina beginning at the level of the pubic symphysis and occurs as discrete lobules within the larger WAT depot (Figure 1b). The pBAT extends cranially along the cervix and terminates at approximately the cervical-uterine junction; some BAT is also found near the base of the bladder. The pBAT lobules are contiguous with or immediately adjacent to the urethra and cervix, and they do not extend laterally through the large WAT depot that envelopes these structures. In males, the pBAT is composed of small lobules in the surrounding WAT that extend cranially along the pelvic urethra, starting at the pubic symphysis, as in the female. The pBAT is also found along the caudal aspect of the ventral prostate, at the base of the bladder and seminal vesicles, and around the seminal colliculus.

**Figure 2.**
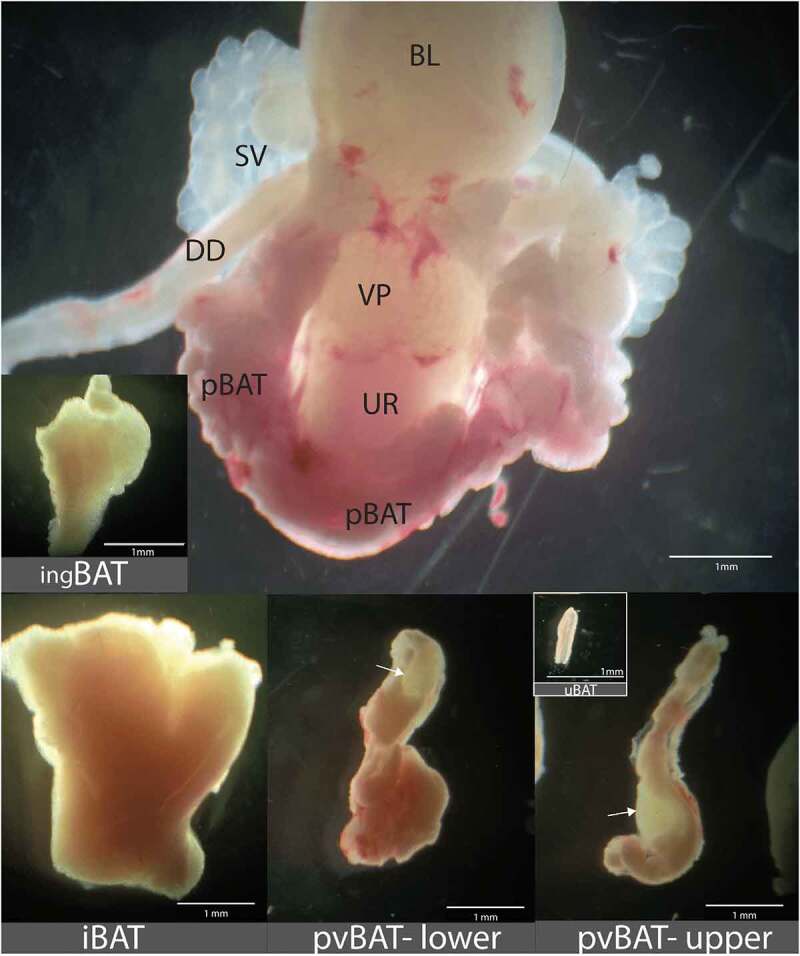
BAT depots visualized using a dissecting microscope with transmitted light and darkfield microscopy. The upper panel shows a ventral view of pBAT in a postnatal day 21 male. The pBAT is seen on both sides of the urethra, but at this early developmental stage there is also a bridge of pBAT on the ventral surface of the urethra connecting the contralateral pBAT on the other side of the urethra. DD = ductus deferens, SV = seminal vesicle, UR = urethra, VP = ventral prostate, BL = bladder. Remaining panels show (counterclockwise) ingBAT, iBAT and lower and upper pvBAT from postnatal day 70 mice; note the sacral lymph node (arrow) in the lower pvBAT and the lumbar lymph node (arrow) encased in the upper pvBAT. uBAT from a postnatal day 5 mouse is shown as an inset in the pvBAT-upper image. Note that the magnification bars in images of the ingBAT, iBAT, lower and upper pvBAT and uBAT and uBAT are all equal, allowing direct comparison of relative BAT depot sizes.

The mouse perivascular BAT (pvBAT) is a thin strand of tissue (Figures 1b, 2) consisting of two anatomically distinct and non-contiguous portions. The more cranial portion of the pvBAT lies (i) in the retroperitoneal space at the level of the descending colon; (ii) lateral to the descending aorta and inferior vena cava, and (iii) on the ventral surface of the psoas minor muscle. In postnatal day 60 mice, the pvBAT originates at approximately the level where the right ovarian vein branches from the caudal vena cava, and envelops the lumbar lymph nodes bilaterally (Figure 1b). At the descending aorta-common iliac artery junction, the cranial pvBAT continues bilaterally along the lateral aspect of the common iliac and then terminates just past where these vessels bifurcate from the descending aorta. The second portion of the pvBAT (Figure 1b) originates along the inferior aspect of the caudal vena cava-common iliac vein junction, where it envelops the sacral lymph node located at the inferior aspect of the bifurcating vessels. This more caudal portion of the pvBAT extends along the medial aspect of the common iliac past where the internal and external iliac branch from the common iliac to finally terminate near the pubic symphysis.

Ureter BAT (uBAT) forms a discontinuous tunic surrounding the ureter (Figures 1b, 2). The uBAT extends from the origin of the ureter below the kidney, where this BAT is continuous with the perirenal BAT, to the termination of the ureter at its juncture with the bladder, although the BAT is less extensive in the middle portion of the ureter compared to the cranial portion near the kidney or caudal portion near the bladder.

The inguinal BAT (ingBAT) is a small pyramidal BAT depot (Figures 1b, 2) located on the ventral side of the mouse’s proximal hindlimb. The ingBAT depot is positioned deep to the larger inguinal WAT depot, but is anatomically distinct from it. The ingBAT depot is situated between the vastus medialis and gracilis muscles of the upper hindlimb and lies cranial to the femoral artery near the branching of the superficial epigastric artery in the region where the abdominal body wall junctures with the upper hindlimb.

### Growth and developmental characteristics of BAT depots

All of the new BAT depots described here are identifiable by postnatal day 4, but the morphology of the various BAT depots changes during subsequent postnatal development. The most striking morphological change with advancing age in the various BAT depots occurs in the pBAT. Although pBAT in adult males occurs only laterally to the urethra and pBAT lobules on the left and right sides of the urethra are anatomically separated (Figure 1b), at postnatal days 4 and 21 pBAT is present not only lateral to the urethra but also extends across the ventral surface of the urethra and is contiguous with the contralateral pBAT (Figure 2). The pBAT on the ventral urethral surface regresses with age, and by adulthood pBAT on the left and right sides of the urethra are separate and anatomically distinct. uBAT is most apparent at the first neonatal age examined, when it is relatively more extensive than at postnatal day 22 or in adulthood. Although pvBAT in adults terminates where the right ovarian vein branches from the caudal vena cava, at postnatal days 4 and 22 it extends all the way to the renal vein, where it is contiguous with the renal BAT.

**Figure 3.**
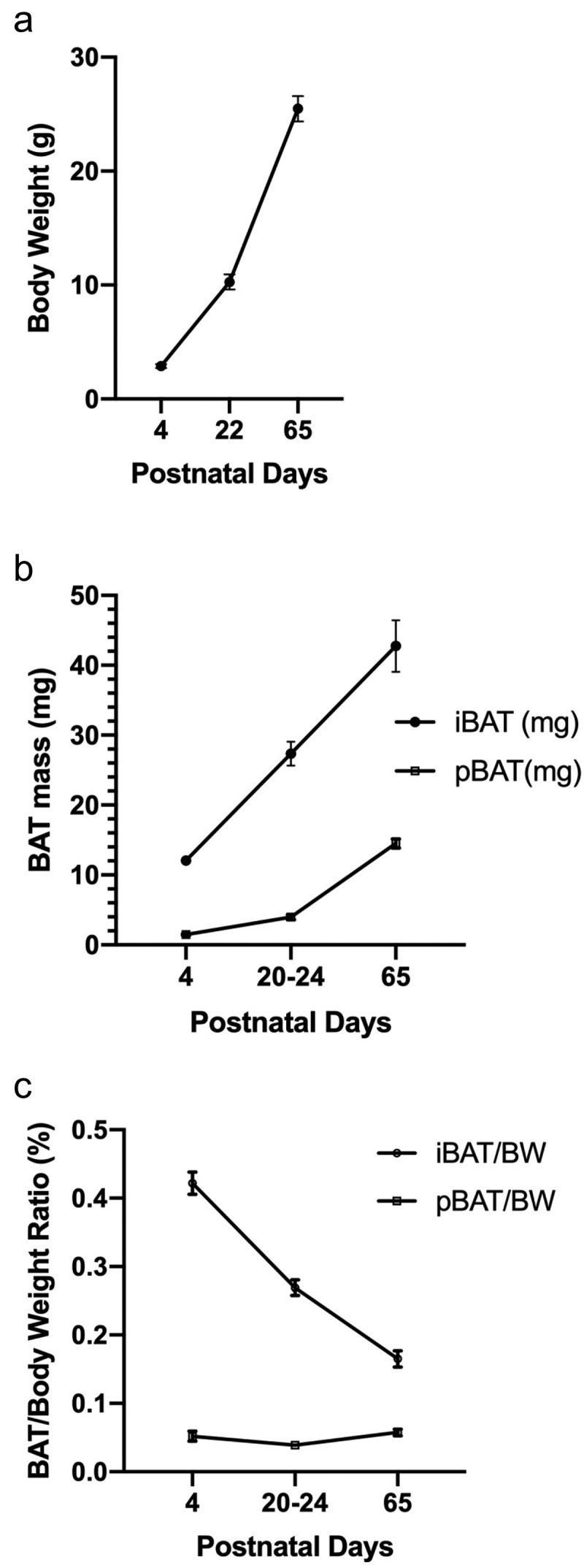
Body weights and absolute and relative weights (BAT weight/body weight; expressed as %) of iBAT and pBAT depots from postnatal days 4–65. In all cases, iBAT and pBAT values represent samples obtained for weighing from one side of the animal only. Data are shown as mean ± SEM, and n = 4–11 for the various ages and BAT depots.

Body weight (BW) and weights of the iBAT and pBAT depots from the neonatal period to adulthood are shown in Figure 3. Both depots increase in weight as the mice age (Figure 3b). The iBAT increases are proportionately less than BW increases, and relative iBAT weight (iBAT weight/body weight) declines during development (Figure 3c). The pBAT is smaller than the iBAT at all ages; pBAT weight is approximately 15% of iBAT at postnatal day 4, but by postnatal day 65, pBAT weighs about 30% as much as iBAT. As a result of this weight increase during development, the pBAT/BW ratio remains relatively constant, in contrast to the decline for iBAT. Weights of pvBAT and ingBAT depots were not determined at all ages, but the postnatal day 22 pvBAT depot weighed 3.8 ± 0.9 mg (n = 4) and the postnatal day 65 pvBAT and ingBAT depots weighed 11.4 ± 1.3 mg and 8.2 ± 1.1 mg (n = 4 for both), respectively. Collectively, the overall weights of the pBAT, pvBAT and ingBAT at day 65 are approximately 75% of that of the iBAT at that age, indicating that these BAT depots could be a significant factor in overall metabolism.

### Expression of UCP1 and induction of UCP1 by an adrenergic agonist in BAT depots

**Figure 4.**
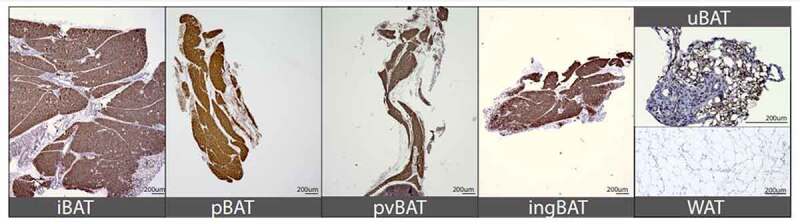
Immunohistochemical staining of UCP1 in various adipose depots. Expression of UCP1 (brown staining) was strong in postnatal 70 iBAT (positive control), as well as in pBAT, pvBAT, ingBAT and uBAT at the same age, but could not be detected in gWAT. Panels A-D are all at the same magnification, and magnification bars for all images are shown in the bottom right hand corner.

Adult iBAT showed intense immunostaining for UCP1, a hallmark of BAT (Figure 4). In contrast, gonadal WAT did not stain for UCP1 (Figure 4). All other new BAT depots identified in this study (pBAT, pvBAT, ingBAT, uBAT) showed the classic histological structure of BAT as well as intense UCP1 immunostaining (Figure 4). The muscularis externa of the ureter also was UCP1-positive at the neonatal, juvenile and adult ages examined (data not shown). Postnatal day 4 pBAT also stained intensely for UCP1 (data not shown), suggesting that BAT depots described here were functional neonatally, when their thermogenic effects are most critical. As an additional control, another tissue that expresses high amounts of mitochondria (kidney) was also immunostained for UCP1. Kidney did not show UCP1 staining despite high mitochondrial expression in this tissue (not shown), indicating that the strong UCP1 staining seen in BAT depots does not result from non-specific effects of high mitochondrial concentrations.

**Figure 5.**
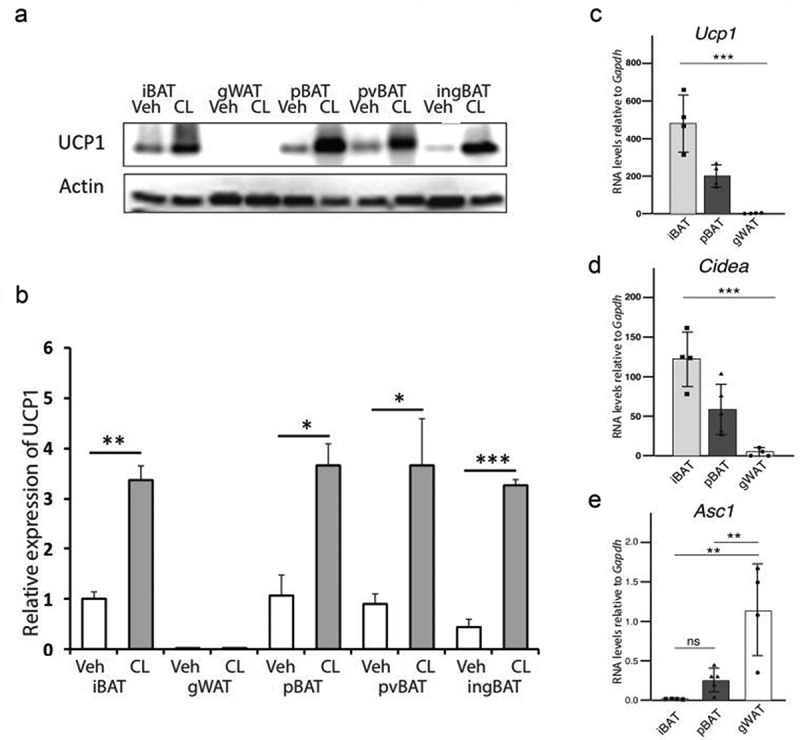
Protein and gene expression are similar in iBAT and the newly identified BATs. Western blotting (a) and quantitation (b) for UCP1 expression in iBAT, pBAT, pvBAT, ingBAT and gWAT in postnatal day 35 female mice given vehicle or the β3-adrenoceptor agonist CL 316,243 (1 mg/kg/day for 3 days). Adipose tissues were collected 24 h after the last injection. The housekeeping protein β actin was used as a loading control. UCP1 expression in all groups was expressed relative to the UCP1 value in vehicle-treated iBAT, arbitrarily set as 1. The n = 3 for all groups used in the Western blotting in (a) and (b) and n = 4 for all groups used in the qPCR studies (d and e). Data are shown as mean ± SEM. *, p < 0.05; **, p < 0.01; ***, p < 0.001.

Western blotting indicated significant baseline UCP1 expression in iBAT, pBAT, pvBAT and ingBAT (Figure 5, a and b). Treatment with the adrenergic agonist CL 316,243 increased UCP1 in all BAT depots. In contrast, UCP1 could not be detected in gonadal (g)WAT following either vehicle or CL 316,243 treatment. The uBAT consists of thin discontinuous strands of BAT that are intimately associated with the ureter and could not easily be separated from it, so western blotting for uBAT was not performed.

### pBAT expresses characteristic BAT, but not WAT, genes

Expression of mRNA for *Ucp1*, alanine serine cysteine transporter-1 (*Asc-1*) and *Cidea* was quantitated in iBAT, pBAT and gWAT (Figure 5c–e). *Ucp1* and *Cidea* mRNA were robustly expressed in iBAT and pBAT, although for both iBAT expression exceeded that in pBAT. *Ucp1* mRNA was essentially undetectable and *Cidea* mRNA was minimal in gWAT (Figure 5). *Zic1* mRNA was abundantly expressed in iBAT. Reduced amounts of *Zic1* mRNA were expressed in pvBAT, but *Zic1* expression was absent in pBAT and ingBAT (data not shown). Conversely, *Asc-1* expression was high in gWAT but minimal in iBAT and pBAT.

### Sympathetic innervation in pBAT

**Figure 6.**
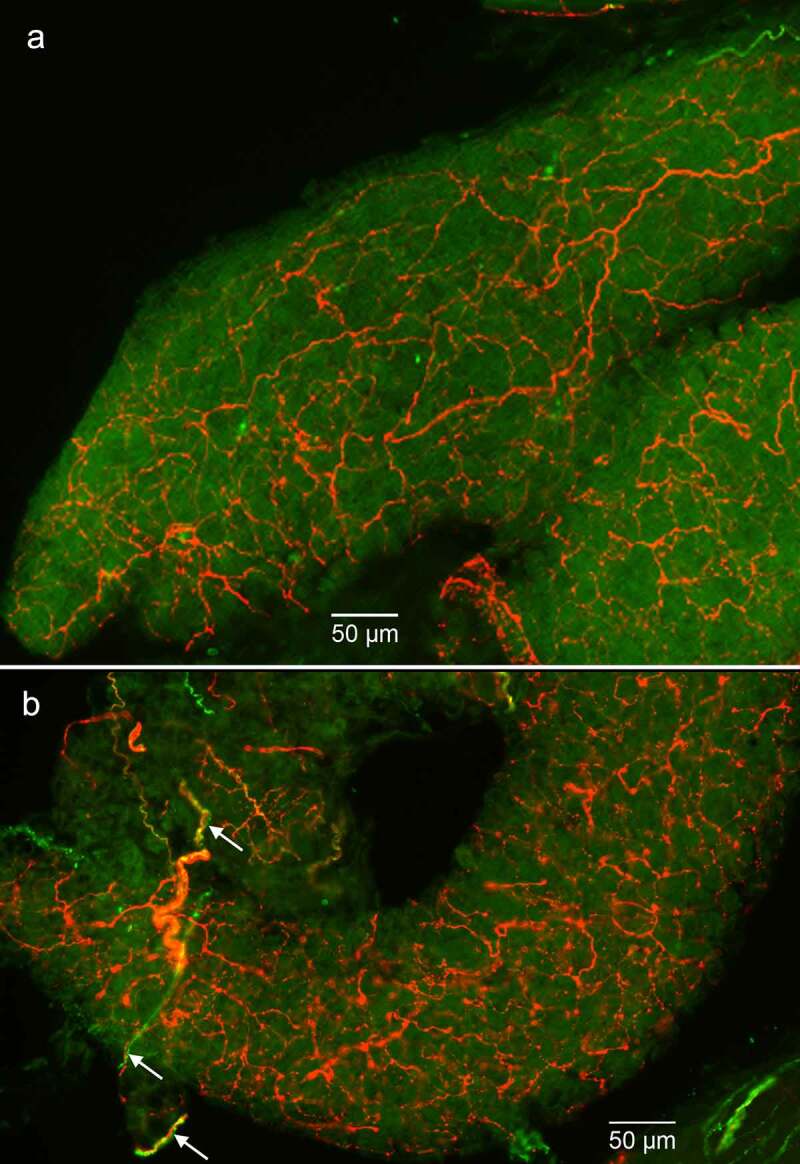
Immunohistochemical fluorescence for TH and CGRP axons in pBAT. (a and b) Tyrosine hydroxylase (TH) expressing sympathetic postganglionic motor axons (red) and CGRP sensory axons (green) in two separate sections of pBAT. As seen clearly in b, the CGRP sensory axons often are located adjacent to TH axons but (i) do not co-label the same axon (white arrows) and (ii) show a different arborization pattern. Images in a and b are flattened Z-stacks. Magnification bars are shown in the bottom of both images.

Tyrosine hydroxylase (TH) is the rate-limiting enzyme found in neurons secreting catecholamine neurotransmitters (i.e. norepinephrine, epinephrine, dopamine) and is typically used as a immunohistochemical marker for sympathetic postganglionic motor neurons and motor axons [20]. There was extensive innervation of pBAT tissue by TH^+^ axons (Figure 6, a and b). Calcitonin gene-related peptide (CGRP) is a marker of peptidergic sensory axons. CGRP^+^ sensory axons were also present in pBAT, although their expression pattern was distinct from the TH^+^ axons (Figure 6b). The TH and CGRP expression noted here are consistent with our identification of these newly described tissues as BAT.

### Transmission electron microscopy (TEM) of iBAT and pBAT

**Figure 7.**
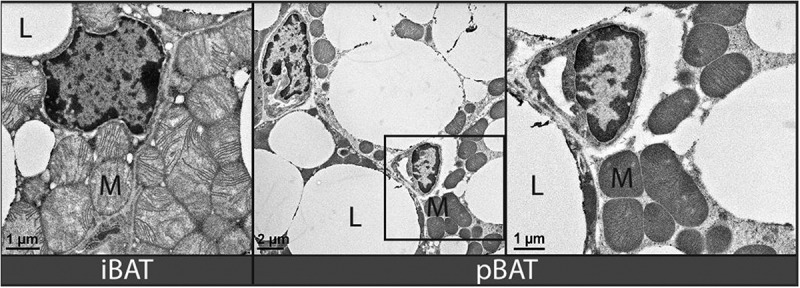
Transmission electron micrographs (TEM) of iBAT and pBAT. Left panel: iBAT had extensive cytoplasmic mitochondria (M) and multilocular lipid droplets (L). Middle panel: lower power view of pBAT showing extensive mitochondria, multilocular lipid droplets and a nuclear morphology that were all similar to iBAT. Right panel: higher power view of pBAT showing a greater magnification of the region highlighted by the square in the middle panel. Magnification bars shown in lower left corner of all images.

Brown adipose tissue is characterized by extensive mitochondria and multilocular lipid droplets. Transmission electron microscopy indicated that these typical BAT features occurred in both iBAT (Figure 7, left panel) and pBAT (Figure 7, middle and right panels). In contrast to WAT, in which the nucleus is highly eccentric due to the single large lipid droplet pushing it to the cell periphery, BAT nuclei are more central, and this was apparent in both iBAT and pBAT.

### pBAT in neonatal rats

To determine whether pBAT also occurred in other species, we examined rats at postnatal day 10. pBAT was readily identifiable in these rats, with a distribution and appearance under darkfield microscopy similar to that in mice. The rat pBAT stained intensely for UCP1 (data not shown), further indicating that this tissue was classical BAT.

## Discussion

Our findings indicate that there are four heretofore undescribed BAT depots in the pelvis, lower abdomen and upper hindlimb of the mouse, anatomical regions where BAT has not been reported previously. pBAT is associated with the male and female reproductive tract in both sexes and with the urethra in the male. uBAT envelops the ureter and the pvBAT lies adjacent to major blood vessels in the lower abdomen and pelvis, whereas the ingBAT is a small depot found between adjacent muscles at the juncture of the upper hindlimb and abdominal body wall. These results indicate that BAT is more widely distributed in this species than previously realized.

All new BAT depots described are present neonatally and then increase in overall size and weight as the animal develops. The weights and sizes of the pBAT and each of the other newly described BAT depots are significantly smaller than iBAT, where the largest amount of BAT is found in the body. However, in aggregate, these four new BAT tissues are comparable in size to the iBAT. Considering their total size in concert with our results showing that these new BAT depots are active metabolically suggests that these BAT depots likely have significant roles in overall BAT function and physiology in the mouse and should be taken into account in studies of BAT activity in this animal.

An important question to consider for all the new BAT depots described is why they were not reported previously. The BAT depots associated with the distal regions of the male and female reproductive tract described here were initially identified using a dissecting microscope with transmitted light and darkfield microscopy, which allowed clear differentiation of BAT from surrounding WAT and was crucial for initial identification of BAT in regions where it was not reported earlier. When viewed under a dissecting microscope with normal brightfield illumination and incident light, the extensive adipose tissue in the urethral/vaginal area in the female and the urethral/prostatic area in the male appears fairly homogeneous and there is little indication that BAT tissue is present. However, under darkfield illumination with transmitted light, the BAT can be easily differentiated from the surrounding WAT tissue, allowing the extent and distribution of the BAT in this region to be clearly visualized.

pBAT is anatomically diffuse and discontinuous and consists of numerous lobular structures and is more structurally similar to subclavicular BAT than the iBAT depot, which is clearly defined anatomically without obvious lobules. Although pBAT volumes appear comparable in males and females, the sexually dimorphic nature of the reproductive organs in this region results in the anatomical distribution of this BAT depot differing in males and females. Whether the thermogenic activity of BAT associated with the male and female reproductive tract has any specific function in these organs is currently unknown.

pvBAT and pBAT are the largest of the new BAT depots described here. At weaning, the pvBAT depot is contiguous cranially with the perirenal BAT and extends bilaterally along the descending aorta and caudal vena cava from just beneath the kidney to the pubic symphysis, following the iliac veins after they branch from the caudal vena cava. Although a mediastinal BAT lying around the thoracic aorta in the thoracic region was described previously [21], the pvBAT described here is anatomically distinct from that thoracic BAT depot. Zhang et al [14] used positron emission tomography-computed tomography (PET/CT) with radioactively labelled compounds to identify areas of intense glucose and fatty acid metabolism corresponding to BAT. These studies suggested intense fatty acid uptake in the midline below the kidneys, especially following β3 adrenergic stimulation, and indicated that there could be BAT in this region. However, this imaging study did not allow the anatomical location of the signal to be precisely determined. Our results indicate that the previously investigated region likely reflects the pvBAT we report here, which is located below the kidneys in the midline.

The pvBAT consists of two thin bilateral strips of tissue. The small diameter of this structure may have impaired detection in previous human and animal studies utilizing tracer methodologies. However, the extensive length of pvBAT, which extends from the renal artery to the pubic symphysis, indicates that it could be physiologically significant. A similar argument can be made for pBAT, which is diffuse and anatomically discontinuous and thus also may be problematical to resolve by current imaging methodologies.

The ingBAT is the only one of the newly described BAT depots that occurs outside the body cavity, although other more peripheral BAT depots such as in the head and neck have been reported [10]. In adults, the size of the ingBAT is less than 25% that of the iBAT, and the ingBAT depot also weighs less then the pBAT depot described here. The ingBAT may be a significant contributor to overall BAT activity, as suggested by previous studies in which increased focal metabolic activity was detected at the anatomical location of the ingBAT. The BAT imaging studies of Zhang et al [14] identified one punctate area of the leg that showed intense metabolism. This activity was ascribed to the presence of beige adipocytes in the inguinal WAT, but the inguinal WAT is much more anatomically diffuse than the sharply delineated signal reported in this study. In the light of the present results, this activity likely emanated from the ingBAT.

The uBAT envelops the ureter and is most pronounced during neonatal and juvenile life. Although it is reduced during adulthood, uBAT around the ureter is still demonstrable. In addition, our results also indicate that the smooth muscle layers of the ureter express UCP1, consistent with a previous report [22]. Because of its close association with the ureter, it is problematic to quantitate the uBAT tissue compared to the other new BAT depots described here. The uBAT that envelops the ureter, especially during neonatal and juvenile life, suggests that this BAT may play a role in ureter development or function, but the specific roles of uBAT in normal ureter physiology, remain to be determined. Work from the early 20th century demonstrated that ligation of the ureter led to bone formation in this tissue [23]. This process, known as heterotopic ossification, has more recently been shown to involve brown adipocytes [24]. Thus, the uBAT described here may be mechanistically involved in the ureter heterotopic ossification following ligation that has previously been documented.

Because uBAT is continuous with the renal BAT cranially and the distal juncture of the ureter is near where pBAT is located at the base of the bladder, all three of these BAT depots may have a common embryological origin, although this remains to be directly addressed.

A hallmark of BAT is UCP1 expression, which is essential for thermogenesis in this tissue [25,26]. Importantly, UCP1 expression in BAT is inducible by cold stress or β3-adrenoceptor agonists such as the endogenous hormone norepinephrine or synthetic ligands such as CL 316,243 that stimulate glucose uptake, heat production and oxygen consumption [22,23]. All of the four newly identified BAT depots robustly express UCP1 by immunohistochemistry, a control gonadal WAT sample does not. Kidney, which has extensive mitochondria, also does not express specific UCP1 staining l Western blotting also showed that UCP1 is detectable in all control BAT depots (without CL 316,243 stimulation). Furthermore, UCP1 is strongly inducible by CL 316,243 in iBAT and all of the new BAT depots reported, with the magnitude of the increases in UCP1 in response to CL 316,243 in the four new BAT depots being similar in magnitude to those in iBAT. UCP1 was not detectable in WAT either before or after stimulation with CL 316,243.

Mestres-Arenes [27] recently described perivascular adipose near the abdominal midline that has a WAT histological appearance with some multilocular adipocytes visible. This tissue expressed UCP1 under thermoneutral conditions, but at a level only about 10% of what is seen in iBAT. Both the intense baseline immunohistochemical staining of our pvBAT, as well as our western blotting results indicating that the pvBAT expresses UCP1 in a basal and CL 316,243-stimulated state, suggest that this tissue is classical BAT, and not the WAT with some BAT characteristics previously described in the area near the kidney.

In addition to UCP1 measured by western blotting, our newly identified BAT depots also show a molecular signature consistent with classical BAT. Expression of *Ucp1* and *Cidea* mRNAs, which are diagnostic for BAT, is high in control iBAT as well as the new BAT depots described here, but absent in WAT. ASC-1, a cell surface marker of WAT that is minimally expressed in BAT [28], is robustly expressed in gWAT, but not in iBAT or the other BAT depots. These results confirm that the BAT depots described here have classic molecular characteristics of BAT, although minor WAT contamination of depots such as the pBAT, which occur in vivo interspersed within WAT, is likely inevitable and may account for the low *Asc-1* mRNA signal in pBAT.

The norepinephrine analog CL 316,243 is a potent and highly selective β3-adrenoceptor agonist that stimulates UCP1 expression in BAT, but not WAT [1]. The marked stimulation of UCP1 by CL 316,243 in all BAT depots, in conjunction with other results presented here, demonstrate that the new BAT depots described express an extensive number of characteristics that are unique to BAT but not seen in WAT.

Both the iBAT and pBAT contain extensive mitochondria and multilocular lipid droplets as demonstrated by TEM. These are classical characteristics of BAT and confirm that pBAT expresses the unique features of BAT [20]. Another classic characteristic of BAT is extensive sympathetic innvervation. The thermogenesis that is the hallmark of BAT is induced by the release of the neurotransmitter noradrenaline from extensive sympathetic fibres present in this tissue and subsequent stimulation of UCP1. Conversely, WAT does not express the extensive sympathetic innervation characteristic of BAT. The rate-limiting step in catecholamine synthesis is regulated by TH, and is typically used as an immunohistochemical marker for sympathetic postganglionic motor neurons and motor axons [20]. The extensive sympathetic innervation seen in pBAT is comparable to that seen in control iBAT sections and far exceeds that in WAT, further establishing pBAT as classical BAT.

CGRP is a neuropeptide that has been previously used as a marker of sensory peptidergic neuronal fibres of interscapular and perirenal BAT and is believed to be involved in BAT thermogenesis, energy metabolism, food intake and lipid homoeostasis [19,29,30]. These actions may be mediated by effects on the morphological characteristics of adipocytes and depots along with mediating inflammatory and metabolic responses [31]. Our results show that pBAT also expresses CGRP. The CGRP sensory axons are much less numerous than the sympathetic axons and often occur in close proximity to the more numerous TH axons. However, CGRP does not co-label the same axons as TH and these axons innervate different areas of the BAT, again consistent with previous findings in iBAT [20].

It has been known for several years that mice can have UCP1-positive (beige) cells in their WAT depots that express UCP1 and resemble BAT histologically [32]. Although numbers of these beige adipocytes can be increased by cold exposure and other means, beige fat has only patchy occurrence of UCP1-expressing cells that show histological characteristics of BAT. These are distributed thoughout the larger tissue that shows WAT characteristics. In contrast, our newly described BAT depots show intense UCP1 immunohistochemical staining and classic BAT histological characteristics throughout and thus are clearly distinct from beige adipose tissue.

The common and unique features of WAT, BAT and beige adipose tissue development have generated extensive interest [21,33–35]. The developing iBAT expresses *Myf5*, a transcription factor involved in muscle differentiation, suggesting iBAT and muscle may be derived from a common embryonic lineage [36]. However, subsequent work indicated that some other BAT depots did not express *Myf5* developmentally, suggesting a different embryonic lineage [37]. Cells with characteristics of brown adipocytes in beige adipose tissue appear to arise from a different developmental lineage and evidence has been presented supporting both de novo differentiation of brown adipocytes [38] as well as transdifferentiation of existing white adipocytes to cells with characteristics of brown adipocytes [39]. Thus, brown adipocytes appear to develop from several different lineages.

Interscapular BAT strongly expresses *Zic1* mRNA, and this was initially suggested as a marker to identify BAT and differentiate it from beige adipose tissue. However, subsequent work indicated that *Zic1* appeared to only be expressed in anterior BAT depots, but was reduced or absent in more posterior BAT depots (reviewed by [34]). In addition, *Zic1* could be transiently expressed in adipocytes undergoing beiging in WAT under some conditions [40].

Our results indicate that *Zic1* mRNA is abundantly expressed in iBAT, in agreement with previous findings. Reduced amounts of *Zic1* mRNA were also expressed in pvBAT, but *Zic1* mRNA expression was absent in the pBAT and ingBAT. These results are consistent with previous findings that *Zic1* mRNA expression in BAT is preferentially expressed in more anterior BAT depots, which may be indicative of different developmental origins for these depots.

Our data indicating that pBAT also occurs in the rat suggest that this BAT depot is not confined to mice but occurs in other species as well. A critical question that remains to be addressed is whether the new BAT depots described here are unique to rodents or occur in humans and other mammals. Imaging studies to examine BAT distribution in humans have shown signalling in the lower abdominal region as well as the pelvic region around the bladder [10,14], although these studies focused on BAT depots in the upper thoracic region. The lack of reports indicating BAT presence in the lower abdominal/pelvic region may have suggested to the authors that the signals detected in this region were non-specific and did not represent BAT, or the signals in this region may simply not have been the focus of the study and thus were not noted. Additional studies are required to determine whether human BAT depots are present in similar anatomic locations to the new rodent depots described here. Nevertheless, the extensive nature of the lower abdominal/pelvic depots in the mouse, the previous suggestions of BAT in these regions from earlier studies and the strong correlation between the anatomical locations of thoracic BAT depots in the mouse and human [14] suggest the new-found BAT may be clinically relevant.

## Methods

### Animals and care

The C57BL/6 mice used in these studies were purchased from Charles River or bred in our colony. Mice were housed in standard polycarbonate/polysulphones cages at 25°C with 12 L:12D cycles and given water and a standard rodent diet ad libitum. All animal experiments were approved by the IACUC of the University of Florida and conducted in accordance with the NIH Guide for the Care and Use of Laboratory Animals.

### Visualization and quantification of BAT during postnatal development

BAT depots were visualized using transmitted light and darkfield microscropy, which allowed these tissues to be differentiated from WAT in the pelvic area and other locations where BAT depots were identified. Images of BAT depots were captured using an Olympus SZH dissecting microscope interfaced with a personal computer. Body weight (BW) and weights of the iBAT and pBAT depots were determined at neonatal (postnatal day 4), juvenile (postnatal day 20–24) and adult (postnatal day 60–65) ages.

### Immunohistochemistry for UCP1

Potential BAT depots from WT mice ranging from postnatal days 4–70 were immunostained for UCP1. iBAT and white adipose tissue (WAT) were used as positive and negative controls for UCP1 expression, respectively. Adult kidney was also used as a negative control. For UCP1 immunohistochemistry, BAT samples were immersion fixed in 10% neutral-buffered formalin for at least 48 h. Samples were paraffin embedded, sectioned at 5–6 μm, deparaffinized, and rehydrated. Antigen retrieval was performed in 10 mM TRIS-EDTA solution (pH 9.0), for 18 min and cooled to ambient temperature. Endogenous peroxidase activity was quenched by incubation with 0.6% H_2_O_2_ in methanol for 20 min. Slides were incubated with a primary rabbit monoclonal IgG antibody for UCP1 (Abcam; Cat# ab208483; RRID:AB 2722676) overnight at a 1:4000 dilution. Binding of primary antibody to UCP1 was localized using the Vectastain ABC and DAB Substrate Kit (Vector Laboratories, Newark, CA), according to manufacturer’s instructions. Sections were counterstained with Gill haematoxylin. Negative controls were processed without primary antibody and counterstained as above. Images were captured using an Olympus Olympus BH2 microscope with PlanApo lenses interfaced with a personal computer.

### Immunofluorescence for tyrosine hydroxylase (TH) and calcitonin gene related peptide (CGRP)

Isoflurane inhalation was used to deeply anesthetize mice and then in vivo cardiac perfusion was performed by first using a PBS buffer (pH = 7.4), followed by a periodate-lysine-2% paraformaldehyde (PLP) fixative. BAT samples were then harvested and fixed for 36 h at 4°C, followed by a 0.1 M PBS (pH 7.6) rinse and immersion for 24 h at 4°C. Samples were then moved to a 30% sucrose solution in 0.1 M phosphate buffer with 0.1% sodium azide solution for two weeks prior to processing.

Using slight variations to our published protocol [41] samples were embedded and cryosectioned at 20 μm (Richard-Allan HM550VP cryostat) and placed onto gelatin and poly-l-lysine subbed glass slides. Liquid IHC blocker was placed around tissue sections, they were covered with a 4% goat serum super block, and then placed in a humidity chamber at room temperature for 1 h. Tissue sections were then incubated at room temperature for 18 h with primary antibodies against Guinea pig CGRP (Fitzgerald Industries International, Cat# 20 R-CP007; RRID:AB_1282813) or chicken TH (EnCor Biotechnology, Inc., Cat #CPCA-TH; RRID:AB_2737416). After incubation with primary antibodies, sections were washed 4 times in a 1% goat serum Triton x100 PBS solution for 10 minutes per wash. Sections were then incubated for 3 h at room temperature with the following secondary antibodies: Alexa Fluor-488 goat anti-Guinea pig and Alexa Fluor-647 goat anti-chicken (Thermo Fisher Scientific, both diluted 1:100), followed by the 4X-wash procedure. Slides were then covered and mounted with ProLong™ Gold Antifade Mount. Images were obtained using a Keyence epi-fluorescence microscope (Keyence BZ-X710).

### Western blotting to assess UCP1 expression in adipose tissue

For UCP1 western blotting, 5–20 mg of iBAT, pBAT, ingBAT, pvBAT and gWAT from postnatal day 35 female mice was lysed in T-PER lysis buffer. Proteins were separated by SDS-PAGE and transferred to nitrocellulose membranes. Blots were probed with the UCP1 antibody used for immunohistochemistry (1:5000) followed by goat anti-rabbit horseradish peroxidase conjugate secondary antibody (BioRad) and incubation in chemiluminescent reagents. Signal was then captured using a LI-COR C-DiGit Chemiluminescence Western Blot Scanner. Actin was used as a loading control, and was visualized using antibody from Santa Cruz Biotechnology at a dilution of 1:2000. Comparative intensity of UCP1 bands was measured by densitometry using NIH Image J software.

### Treatment with CL 316,243

Two groups of five female WT C57BL/6 female mice (postnatal day 35) were given 3 daily ip injections with the adrenergic agonist CL 316,243 (Cayman Chemical; 1 mg/kg BW) in 0.1 ml vehicle or vehicle alone (controls). Samples (approximately 10 mg) of gonadal WAT were collected 24 h after the final treatment and frozen either with liquid nitrogen for subsequent PCR analysis or on dry ice for western blotting. iBAT, pBAT, pvBAT and ingBAT (5–10 mg each) were then harvested for western analysis. Additional iBAT and pBAT samples from control mice were used for PCR.

### Quantitative RT-PCR (qPCR)

Total RNA was isolated with TRI® reagent (Sigma-Aldrich) following the manufacturer’s recommendations and treated with DNase I (Roche Diagnostics Corporation), according to the vendor’s instructions. DNase I-treated RNA was purified with Qiagen Mini columns (Qiagen), and the quantity and quality of RNA were determined spectrophotometrically with a NanoDrop Lite spectrophotometer. Equal concentrations of total RNA were reverse transcribed using an M-MLV (Moloney Murine Leukaemia Virus) Reverse Transcriptase kit (Invitrogen, Thermo Fisher Scientific), following the manufacturer’s specifications. Quantitative RT-PCR experiments were performed in a Roche LightCycler480 (Roche Life Science) using SYBR Green PCR master mix (Applied Biosystems).

For the analysis of gene expression, standardization was performed relative to *Gapdh* RNA. The samples were analysed in triplicate from at least four biological replicates (animals), and the fold change was calculated using the ∆∆Ct method. Statistical analysis (one-way ANOVA) was performed on the ∆∆Ct values, and the results were considered significant at P < 0.05. Significance between groups was determined by the Holm-Sidak multiple comparisons post hoc test. Results were graphed as RNA levels relative to a *Gapdh* control using GraphPad Prism (6.02 version) software.

### Transmission electron microscopy (TEM) of pBAT and iBAT

Samples of pBAT and iBAT from postnatal day 70 males were dissected and cut into 1 mm^2^ pieces that were immediately placed in a fixative of 4% paraformaldehyde + 2.5% glutaraldehyde in 0.1 M sodium cacodylate, pH 7.24, and kept in 4°C for 48 hours. Fixed samples were the placed in fresh fixative followed by several buffer washes with 0.1 M sodium cacodylate, pH 7.24, post-fixed with buffered 2% OsO_4_, water washed and dehydrated in a graded ethanol series (25–100% in 5% increments) followed by 100% acetone. Dehydrated samples were infiltrated with Embed/Araldite epoxy resin and Z6040 embedding primer (EMS) and cured for 72 hours at 65°C. Ultra-thin sections were collected on carbon coated Formvar 100 mesh copper grids (EMS) and post-stained with 2% aqueous uranyl acetate and Reynold’s lead citrate. Sections were examined with a FEI Tecnai G2 Spirit Twin TEM (FEI Corp.) and digital images were acquired with a Gatan UltraScan 2k × 2k camera and Digital Micrograph software (Gatan Inc.) at the University of Florida, ICBR Electron Microscopy Core.

### Statistical analysis

Data were analysed using the Student t-test to identify differences between two groups. When comparing more than two groups, one-way analysis of variance (ANOVA) followed by the Dunnett multiple comparisons test was used. Differences were considered significant at p < 0.05. Statistical analysis was performed with GraphPad Prism 6.0 (GraphPad Software, Inc., San Diego, CA).

## Summary sentence

Several new brown adipose tissue depots are reported in the pelvic and lower abdominal region of mice, anatomical areas where this tissue has not been described previously.
